# Biosynthesis of gold nanoparticles assisted by the intracellular protein extract of *Pycnoporus sanguineus* and its catalysis in degradation of 4-nitroaniline

**DOI:** 10.1186/s11671-015-0856-9

**Published:** 2015-03-25

**Authors:** Chaohong Shi, Nengwu Zhu, Yanlan Cao, Pingxiao Wu

**Affiliations:** School of Environment and Energy, South China University of Technology, Guangzhou, 510006 China; The Key Laboratory of Pollution Control and Ecosystem Restoration in Industry Clusters of Ministry of Education, Guangzhou, 510006 China

**Keywords:** Gold nanoparticles, Intracellular protein extract, *Pycnoporus sanguineus*, Biosynthesis, Catalysis

## Abstract

The development of green procedure for the synthesis of gold nanoparticles (AuNPs) has gained great interest in the field of nanotechnology. Biological synthetic routes are considered to be environmentally benign and cost-effective. In the present study, the feasibility of AuNPs’ synthesis via intracellular protein extract (IPE) of *Pycnoporus sanguineus* was explored. The characteristics of generated particles of formation, crystalline nature, and morphology and dimension were analyzed by UV-vis spectroscopy, X-ray diffraction (XRD) and transmission electron microscopy (TEM), respectively. UV-vis spectra exhibited strong absorption peaks in 520 to 560 nm, indicating the formation of AuNPs. XRD analysis revealed that the formed AuNPs were purely crystalline in nature. TEM observation showed that AuNPs with various shapes including spherical, pseudo-spherical, triangular, truncated triangular, pentagonal, and hexagonal, ranging from several to several hundred nanometers, were synthesized under different conditions. The average size of AuNPs decreased from 61.47 to 29.30 nm as the IPE addition increased from 10 to 80 mL. When the initial gold ion concentration changed from 0.5 to 2.0 mM, the average size rose from 25.88 to 51.99 nm. As in the case of solution pH, the average size was 84.29 nm with solution pH of 2.0, which diminished to 6.07 nm with solution pH of 12.0. Fourier transform infrared (FTIR) analysis implied that the functional groups including hydroxyl, amine, and carboxyl were involved in the reduction of gold ions and stabilization of AuNPs. The catalysis results showed that 0.019 mg of AuNPs with average size of 6.07 nm could catalyze the complete degradation of 12.5 μmol of 4-nitroaniline within 6 min and the degradation rate increased drastically with the addition of AuNPs. All the results suggested that the IPE of *P. sanguineus* could be potentially applied for the eco-friendly synthesis of AuNPs.

**Electronic supplementary material:**

The online version of this article (doi:10.1186/s11671-015-0856-9) contains supplementary material, which is available to authorized users.

## Background

Nanotechnology is becoming the focus of scientific researches all over the world during the last few decades. The synthesis of noble metal nanoparticles with controlled morphology and dimension exhibits outstanding importance in this field, as metal particles at nanoscale possess many properties significantly different from those of the corresponding bulk materials [1]. Particularly, gold nanoparticles (AuNPs) are of the most importance owing to their wide applications in optics, electronics, catalysis, and biomedicine [2,3].

Various physical and chemical approaches are available to synthesize AuNPs [1], while almost all those methods are energy or capital intensive. What is more, many toxic chemicals including sodium borohydride (NaBH_4_), surfactants, or thiol groups are involved, thus precluding the applications of the produced AuNPs in many clinical and biological areas. Therefore, the development of a clean, cost-effective, and environmentally benign process to synthesize AuNPs is in urgent demand.

Currently, a lot of biological materials such as bacteria, fungi, algae, and plants have been reported to synthesize AuNPs [4-7]. Most researches focused on biosynthesis of AuNPs with the whole cells, and the AuNPs were deposited on the cell wall or in the cytoplasmic region [4,8]. Cell-free spent medium or cell-free extract, containing the functional substances including secreted metabolites or eluted extracellular polymeric substances, were also applied to synthesize AuNPs [9,10]. As we know, fungi are able to secrete more proteins, produce more biomass, and show higher metal tolerance and bioaccumulation ability [11]. So far, many fungal species such as *Rhizopus oryzae* [11], *Neurospora crassa* [12], *Trichoderma harzianum* [13], *Aspergillus oryzae* [9], *Helminthosporum solani* [14], *Fusarium semitectum* [15], and *Candida albicans* [16] have been reported to successfully synthesize AuNPs either through extra- or intracellular manners. Thus, fungi are considered to be the most promising candidates for AuNPs synthesis.

The catalytic property of AuNPs is widely concerned from the standpoint of application, especially in the case of degrading contaminants in wastewater. Previous reports have revealed that AuNPs synthesized in biological systems could effectively catalyze the degradation of 4-nitrophenol [2,17]. The biosynthesized AuNPs showed much better catalytic activity than chemically synthesized AuNPs [11]. In addition, biosynthesized AuNPs could even be reused for many rounds [18].

In the present study, for the first time, we used the intracellular protein extracts (IPE) of a white rot fungal strain *Pycnoporus sanguineus* (*P. sanguineus*) to synthesize AuNPs. It was reported that *P. sanguineus* could produce large amount of reductase such as laccases, which might enable us to synthesize AuNPs by the fungus [19,20]. The effects of reaction parameters including IPE addition, initial gold ion concentration, and solution pH on the characteristics of synthesized AuNPs were evaluated. The catalytic activity of biosynthesized AuNPs was investigated in degradation of 4-nitroaniline (4-NA).

## Methods

## Materials

The reagents used were of analytical grade. Chloroauric acid (HAuCl_4_°3H_2_O), 4-NA, NaBH_4_, dextrose, KH_2_PO_4_, MgSO_4_ · 7H_2_O and other chemicals were purchased from Aladdin, Shanghai, China. Chloroauric acid was dissolved in ultrapure water to prepare stock solution for further use.

### Fungal strain, growth conditions, and preparation of IPE

The fungal strain *P. sanguineus* (CGMCC 5.00815) was purchased from China General Microbiological Culture Collection Center, maintained on comprehensive potato dextrose agar (PDA) slants, and kept in refrigerator for further use. To obtain biomass for AuNPs synthesis, *P. sanguineus* was inoculated into nutrition liquid medium, followed by incubation on a rotary shaker at 165 rpm and 25°C. After 3 days of fermentation, the biomass was harvested by centrifugation (10,000 rpm, 4°C, and 20 min) and then washed thoroughly with sterile ultrapure water for three times. To prepare IPE, 10 g of biomass (wet weight) was re-suspended in sterile water and subsequently sonicated using a SONICS VCX150 ultrasonic cell disruptor for 10 min at 60% amplitude. The cell debris was removed by centrifugation, and the supernatant was diluted to 50 mL, which was donated as IPE.

### Synthesis of AuNPs

Biosynthesis of AuNPs was carried out by mixing specific volume of IPE and HAuCl_4_ stock solution without any other extraneous chemicals. The influences of reaction conditions including IPE addition, initial gold ion concentration, and solution pH were evaluated. To check the effect of IPE addition, 10, 20, 40, and 80 mL of IPE were respectively mixed with HAuCl_4_ aqueous solution, and the mixture was diluted to 100 mL with the final gold ion concentration of 1 mM. Amount of 80 mL of IPE was mixed with HAuCl_4_ aqueous solution with the final gold ion concentration of 0.5, 1.0, 1.5, and 2.0 mM to investigate the effect of initial gold ion concentration. As for the effect of solution pH, 80 mL of IPE was mixed with HAuCl_4_ aqueous solution with the final concentration of 1.0 mM, and the pH was adjusted with 0.1 M NaOH or HCl to 2.0, 4.0, 6.0, 8.0, 10.0, and 12.0. The mixtures above were incubated at 165 rpm and 30°C. The formation of AuNPs was monitored by visual inspection of the color change and measuring the UV-vis spectra of the reaction mixture.

### Characterization of AuNPs

The formation of AuNPs was further confirmed by UV-vis spectroscopic measurement of the reaction mixture solution, which was performed on a Shimadzu UV-2450 spectrophotometer (Shimadzu Corp., Kyoto, Japan) (range 400 to 700 nm) with ultrapure water as the reference, and operated at a resolution of 1 nm. X-ray diffraction (XRD) analysis of the biogenic AuNPs was done on a Bruker D8 ADVANCE X-ray diffractometer (Bruker Corp., Billerica, MA, USA) equipped with Cu Kα radiation (*λ* = 0.154 nm), operated at a voltage of 40 kV and a current of 40 mA. The samples for XRD analysis were prepared by dropping the reaction solutions on glass substrates, followed by air drying. Analysis was made between the 2*θ* ranges of 20° to 90° with a step size of 0.02°. The transmission electron microscopic (TEM) study was carried out to determine the morphology and dimension of the synthesized AuNPs, which was conducted on a Hitachi-7650 instrument (Hitachi Ltd., Tokyo, Japan) operated at an accelerating voltage of 80 kV. The samples for TEM study were prepared by dropping two or three drops of the reaction solution onto carbon-coated copper TEM grids. The extra fraction was removed using a blotting paper and then dried at room temperature. Fourier transform infrared (FTIR) spectroscopic measurement was carried out to identify the potential functional groups responsible for the reduction of gold ions and stabilization of the synthesized AuNPs, which was recorded on a PerkinElmer 1725X FTIR spectrometer. The AuNPs were collected by centrifugation (12,000 rpm, 4°C, and 20 min) and subsequently washed three times to remove free proteins or other compounds present in the reaction solution, followed by vacuum freeze-drying. The original IPE was also vacuum freeze dried and then subjected to FTIR measurement.

### Catalytic application of AuNPs in 4-NA degradation

The degradation of 4-NA by NaBH_4_ was studied as a model reaction to explore the catalytic activity of biosynthesized AuNPs. Typically, 1 mL of AuNPs solution (average size of 6.07 nm, 0.19 mg/mL) was added to a flask containing 25 mL of 0.5 mM 4-NA solution and 25 mL of 50 mM NaBH_4_ solution to trigger the catalytic reaction. Blank control without the addition of AuNPs was also conducted. The degradation of 4-NA was monitored by measuring the UV-vis spectra of the reaction mixture at a time interval of 3 min in the range of 250 to 500 nm. The decrease in the absorption peak centered at 380 nm with time indicated the reduction of 4-NA, and the increase in the absorption peak at about 300 to 310 nm indicated the formation of the corresponding reaction product of *p*-phenylenediamine (*p*-PDA). Different volumes of AuNPs solution were added to the above mixture to evaluate the effect of catalyst addition on the degradation process.

## Results and discussion

### Visual inspection of AuNPs’ formation

On mixing IPE with HAuCl_4_ aqueous solution, the color of the mixtures changed gradually from pale yellow to brown, purple, pink, or bluish violet under different reaction conditions, visually showing the formation of AuNPs. The intensity of the colors increased with time, then reached saturation, and remained unchanged even after 2 months at room temperature, indicating that the synthesized AuNPs were very stable and no aggregation phenomenon occurred. The stability of the AuNPs was likely to be due to the capping of some organic compounds such as proteins present in the IPE. It was reported that proteins can bind to metal nanoparticles through free amine groups or cysteine residues and via electrostatic attraction of negatively charged carboxyl or carbonyl groups, forming a coat covering the particles to prevent agglomeration, then leading to the stabilization of AuNPs [9]. Previous researches revealed that AuNPs exhibited many vivid colors in solution, and the colors were highly dependent on the morphology and size of AuNPs [21]. The vivid colors appeared due to the excitation of surface plasmon vibrations in the AuNPs [22], which was an intrinsic property of metal nanoparticles.

### UV-vis spectroscopy

**Figure 1:**
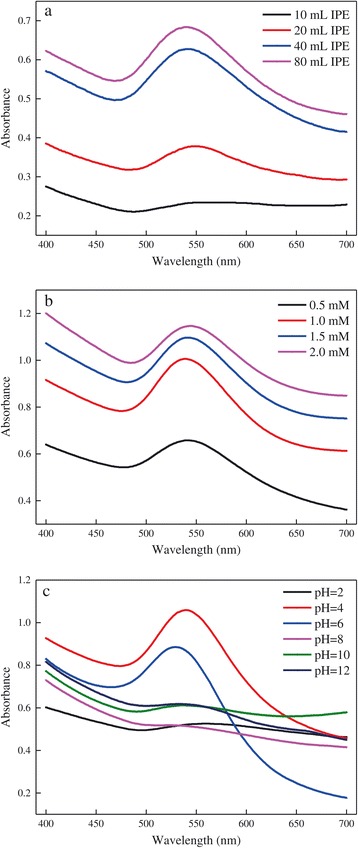
**UV-vis spectra of the reaction mixtures recorded after 24 h of incubation under different conditions. (a)** Effect of IPE addition. **(b)** Effect of initial gold ion concentration. **(c)** Effect of solution pH.

UV-vis spectroscopy has been widely considered to be a useful technique to ascertain the formation of AuNPs [21]. Figure 1 depicted the UV-vis spectra of the reaction mixtures obtained after 24 h of incubation under various conditions. Strong absorption peaks located in the range of 520 to 560 nm were observed, further confirming the formation of AuNPs with various morphology and dimension, which was in agreement with the previous report [23]. The effect of IPE addition was shown in Figure 1a. It can be seen that the intensity of maximum absorption increased with IPE addition, indicating higher AuNPs production, since the intensity was directly proportional to the density of AuNPs [16]. In addition, a blue shift of the maximum absorption was observed as the IPE addition increased, which might be attributed to the formation of AuNPs with smaller size, further verified by TEM study. Husseiny et al. [24] reported that the wavelength of maximum absorption tended to red shift as the particle size increased, which was in accordance with our results. Figure 1b illustrated the spectra of reaction mixtures with different initial gold ion concentrations. The intensity and wavelength of maximum absorption increased along with the increase of initial gold ion concentration, meaning higher production and larger particle size of AuNPs. No distinct regularity was detected with the effect of solution pH on the spectra as shown in Figure 1c, and the highest intensity was obtained with the solution pH of 4.0, while the reasons needed further research.

### XRD analysis

**Figure 2:**
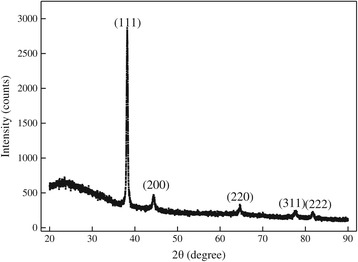
**Representative XRD pattern of biosynthesized AuNPs.** Condition: 80 mL IPE, 1 mM, pH = 2.8.

XRD analysis was performed to study the crystalline structure of the synthesized AuNPs. Figure 2 showed the representative XRD pattern, in which five diffraction peaks at about 2*θ* = 38.2, 44.6, 64.9, 77.8, and 81.7 indexed as the (111), (200), (220), (311), and (222) lattice planes of the standard face-centered cubic phase of metallic gold were observed, further indicating the formation of crystalline AuNPs. No impure peaks were detected, revealing the high purity of the formed AuNPs. The diffraction peak corresponding to the (111) phase was overwhelmingly stronger than the rest of the peaks, suggesting that (111) was the primary orientation [25]. Reaction conditions exhibited very little influences on the XRD patterns of the synthesized AuNPs, although there were slight shifts in the diffraction peak positions, which was a common feature of the biosynthesized nanoparticles [26].

### TEM study

**Figure 3:**
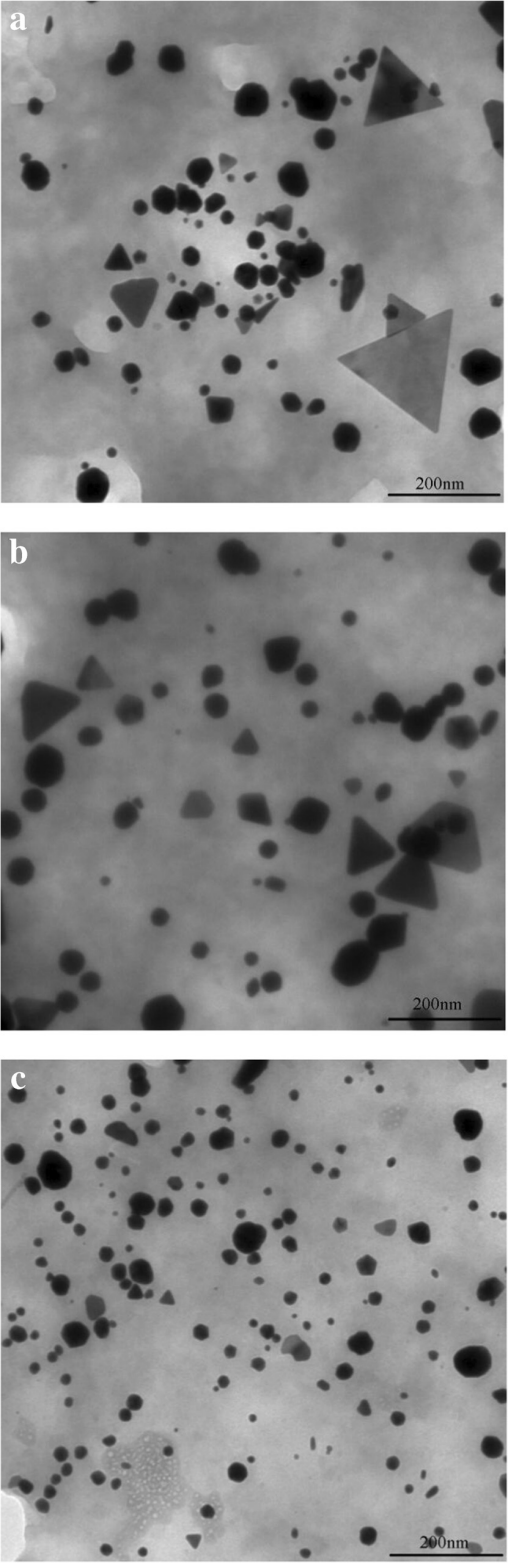
**Representative TEM images of AuNPs obtained under different conditions. (a)** 40 mL IPE, 1.0 mM, pH = 2.7. **(b)** 80 mL IPE, 1.5 mM, pH = 2.5. **(c)** 80 mL IPE, 1.0 mM, pH = 4.0.

The morphology and size of biosynthesized AuNPs were estimated by TEM study. Figure 3 showed the representative TEM images of AuNPs obtained under different conditions, which illustrated the formation of AuNPs with various shapes including spherical, pseudo-spherical, triangular, truncated triangular, pentagonal, and hexagonal. The effects of IPE addition on the characteristics and particle size distributions of as synthesized AuNPs were depicted in Additional file 1: Figures S1 and S2. It can be observed that most spherical or pseudo-spherical AuNPs were relatively smaller compared with triangular, pentagonal, or hexagonal ones. With 10 mL IPE addition, many AuNPs with triangular or hexagonal shapes in the size of 100 to 500 nm were synthesized and the average size was measured to be 61.47 nm. As the IPE addition increased, the formed AuNPs exhibited narrower size distribution and smaller average particle size. When the IPE addition was increased to 80 mL, more than 90% of the AuNPs were smaller than 50 nm, almost 78% were in the range of 10 to 40 nm, and the average particle size decreased to 29.3 nm.

The impacts of initial gold ion concentration were also concerned (Additional file 1: Figures S3 and S4). When the initial concentration was 0.5 mM, AuNPs were mostly spherical or pseudo-spherical with a few triangular, more than 95% of the AuNPs were smaller than 50 nm, and the average size was 25.88 nm. As the initial concentration increased, many truncated triangular, triangular, pentagonal, or hexagonal AuNPs appeared and the average particle size increased as well. Briefly speaking, the average particle size decreased with the increase of IPE addition and increased with increasing initial gold ion concentration. It has been well accepted that gold ion was firstly reduced to form a gold atom, being a nucleus, and the latter formed gold atoms potentially aggregate to this nucleus, thus assembling a gold nanoparticle. With higher initial gold ion concentration, there was greater accessibility of the latter formed gold atoms to the nucleus, obtaining larger AuNPs [27]. Das et al. [5] reported the protein-mediated synthesis of multi-shaped AuNPs, which emphasized the prominent effect of the ratio of gold ion concentration to cell-free extract, and the particle diameter increased with this ratio.

Solution pH also exhibited great influence on the characteristics and particle size distributions of the AuNPs (Additional file 1: Figures S5 and S6). With the solution pH of 2.0, AuNPs ranging from several nanometers to more than 200 nm were produced and the average particle size was measured to be 84.29 nm. As the solution changed to neutral or basic, AuNPs with much smaller and uniform size were synthesized, especially in the case of initial pH of 12.0, more than 98% of the AuNPs were smaller than 12 nm, and 90% were in 4 to 8 nm. It has been reported that reduction rate greatly affected the morphology and size of AuNPs, and slow reduction rate favored the formation of anisotropic and larger AuNPs [10]. During the synthesis of AuNPs with various solution pHs in the present research, the reaction mixture changed to bluish violet in 2 h with solution pH of 12.0. While pink color appeared after 4 h with pH of 6.0 and in the case of pH of 2.0, brown color appeared after 8 h, implying that the reaction rate increased with increasing pH, which was indirectly verified by the particle size distribution. He et al. [28] regarded solution pH as the most important parameter controlling the size and shape of AuNPs. With the solution pH of 7.0, most particles were spherical in 10 to 20 nm, and many triangular AuNPs in 50 to 400 nm were produced as the pH decreased to 4.0. Our previous research (under submission) also revealed that solution pH had noticeable impact on the location and dimension of the biosynthesized AuNPs.

### FTIR spectroscopy measurement

**Figure 4:**
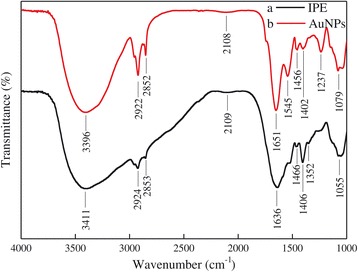
**FTIR spectra of the original IPE (a) and AuNPs (b).**

FTIR spectroscopic studies were carried out to identify the possible functional groups of the biomolecules present in the IPE involved in the reduction of gold ions and stabilization of synthesized AuNPs. The original IPE showed intense and obvious absorption bands at 3,411, 2,924, 2,853, 2,109, 1,636, 1,466, 1,406, 1,055 cm^−1^ (Figure 4a). The strong absorption band at 3,411 cm^−1^ could be referred to the stretching vibration of O-H bond present in carbohydrates or proteins. The bands at 2,924 and 2,853 cm^−1^ were due to aliphatic C-H stretching vibration of carbohydrates. The bands at 1,636 and 1,055 cm^−1^ corresponded to the amide I and carbonyl stretching vibrations in the amide linkage [3]. The band at 2,109 cm^−1^ could be assigned to the nitrogen compounds containing triple or cumulative double bonds such as nitriles (-CN) and cyanates (-O-CN) [16]. The band at 1,466 cm^−1^ might be ascribed to the methylene scissoring vibration from the proteins. The band at 1,406 cm^−1^ was confirmed to the COO^−^ symmetric stretching vibration from the carboxyl side groups in amino acid residues of proteins [11]. Another weak band at 1,352 cm^−1^ assigned to amide III was also observed. The positions of above bands were very close to those reported previously for native proteins [11].

After synthesis of AuNPs (Figure 4b), the O-H peak at 3,411 cm^−1^ shifted to 3,396 cm^−1^. The amide I and carbonyl stretching peaks shifted to 1,651 and 1,079 cm^−1^ respectively, and the band of methylene shifted to 1,456 cm^−1^ as well. The weak band of amide III disappeared and two intense bands centered at 1,545 and 1,237 cm^−1^ corresponded to amide II and C-N stretching vibrations from amines appeared. It was reported that AuNPs could interact with proteins through free amine groups or cysteine residues via electrostatic attraction of negatively charged carboxyl or carbonyl groups, forming a coat covering the particles to prevent agglomeration, thus leading to the stabilization of AuNPs [9]. Such results demonstrated that hydroxyl, amine, and carboxyl groups played important roles in the reduction process and stabilization of synthesized AuNPs. Several reports have also revealed similar conclusions. For instance, Lin et al. reported that -OH and -NH_2_ present on the cell surface of *Pichia pastoris* were involved in the absorption and reduction of gold ions [18]. Ogi et al. reported the participation of carbonyl group in *Shewanella algae* extract during the formation of AuNPs [29].

### Catalytic activity of AuNPs in 4-NA degradation

Along with the rapid development of human society, many organic contaminants have been released into the environment. Among these contaminants, nitroaromatic compounds (NAC) are considered to be highly toxic as they exhibit serious carcinogenic and mutagenic threats to human health [30]. A promising process to eliminate NAC is to transform them into valuable amines aromatic compounds (AAC) through catalytic reduction by NaBH_4_ [31]. In this study, we explored the catalytic reduction of 4-NA to *p*-PDA with NaBH_4_ as reducing agent using biosynthesized AuNPs as catalyst. The reaction was monitored by visual inspection and using the UV-vis spectroscopy, as aqueous of 4-NA exhibited a vivid yellow color and maximum absorption at about 380 nm. Aqueous *p*-PDA was colorless and showed maximum absorption at about 300 to 310 nm [32].

**Figure 5:**
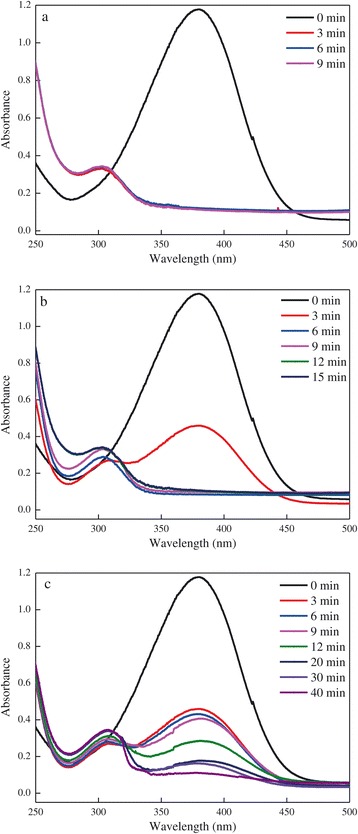
**UV-vis spectra during the degradation of 4-NA catalyzed by different volumes of AuNPs solution. (a)** 1 mL. **(b)** 0.1 mL. **(c)** 0.01 mL.

Upon adding 1 mL of AuNPs solution (average size of 6.07 nm, 0.19 mg/mL) to the mixture, the color faded from yellow to colorless within 3 min. The absorption peak at 380 nm disappeared, and another absorption peak at 302 nm occurred as well (Figure 5a), indicating the rapid catalytic reduction of 4-NA. In contrast, the blank control without the addition of AuNPs showed no change in color and absorption peak even after several days, thus implying the required role of AuNPs in this reduction process, which was in accordance with many previous reports [2,11]. The effect of catalyst addition on the degradation process was also evaluated by varying the volume of AuNPs solution while keeping the other parameters constant. As shown in Figure 5, the reduction rate increased with the increasing addition of AuNPs. The time needed to completely degrade 4-NA was 3, 6, and 40 min in the cases of 1, 0.1, and 0.01 mL of AuNPs solution, respectively. Taking the reduction efficiency and catalyst addition into consideration, 0.1 mL was preferred and about 0.019 mg AuNPs (dry weight) could catalyze the complete reduction of 12.5 μmol of 4-NA in 6 min. Since the concentration of NaBH_4_ much exceeded than that of 4-NA (100-fold), the kinetic reduction was considered to be pseudo-first order. The logarithm of the absorbance of 4-NA at 380 nm (lnA) will then decrease linearly with reaction time, and the calculated slope could be the rate constant (*k*) of the reaction. In this study, the *k* value was calculated to be 0.065 min^−1^.

## Conclusions

In this study, IPE of *P. sanguineus* as reducing and stabilizing agents was used to successfully synthesize AuNPs with various shapes and dimensions. The synthetic process was simple, clean, and could be an alternative to existing physical and chemical methods. Visual inspection, UV-vis spectroscopic measurement, XRD analysis, and TEM observation confirmed the formation and characteristics of AuNPs, which was highly affected by reaction conditions. FTIR analysis implied the interaction between AuNPs and protein extract by functional groups including hydroxyl, amine, and carboxyl, which were possibly responsible for the reduction of gold ions and stabilization. The biosynthesized AuNPs could effectively catalyze the degradation of 4-NA, and 0.019 mg of AuNPs with average size of 6.07 nm was optimized to completely degrade 12.5 μmol of 4-NA in 6 min. It was suggested that the IPE of *P. sanguineus* could be a potential medium to synthesize AuNPs with high catalytic activity in degradation of organic contaminants.

## Additional file

Additional file 1: Figure S1. TEM images of AuNPs synthesized with different IPE additions. **Figure S2.** Particle size histograms of AuNPs synthesized with different IPE additions (measured more than 300 nanoparticles). **Figure S3.** TEM images of AuNPs synthesized with different initial gold ion concentrations. **Figure S4.** Particle size histograms of AuNPs synthesized with different initial gold ion concentrations (measured more than 300 nanoparticles). **Figure S5.** TEM images of AuNPs synthesized with different initial solution pHs. **Figure S6.** Particle size histograms of AuNPs synthesized with different initial solution pHs (measured more than 300 nanoparticles).

## Abbreviations

4-NA: 4-nitroaniline
AuNPs: gold nanoparticles
FTIR: Fourier transform infrared
IPE: intracellular protein extract
NaBH4: sodium borohydride
*p*-PDA: *p*-phenylenediamine
*P. sanguineus*: *Pycnoporus sanguineus*
TEM: transmission electron microscopy
XRD: X-ray diffraction
