# Supplementary material for: Biosynthesis of gold nanoparticles assisted by the intracellular protein extract of Pycnoporus sanguineus and its catalysis in degradation of 4-nitroaniline
Source: Nanoscale Res Lett. 2015 Mar 25;10:147. doi: 10.1186/s11671-015-0856-9 (PMC4385306; doi:10.1186/s11671-015-0856-9)
Supplement: Additional file 1: Figure S1. — TEM images of AuNPs synthesized with different IPE additions. Figure S2. Particle size histograms of AuNPs synthesized with different IPE additions (measured more than 300 nanoparticles). Figure S3. TEM images of AuNPs synthesized with different initial gold ion concentrations. Figure S4. Particle size histograms of AuNPs synthesized with different initial gold ion concentrations (measured more than 300 nanoparticles). Figure S5. TEM images of AuNPs synthesized with different initial solution pHs. Figure S6. Particle size histograms of AuNPs synthesized with different initial solution pHs (measured more than 300 nanoparticles). [file 11671_2015_856_MOESM1_ESM.doc]

**Supplementary Material**

**Biosynthesis of gold nanoparticles assisted by the intracellular protein extract of *Pycnoporus sanguineus* and its catalysis in degradation of 4-nitroaniline**

Chaohong Shi^1^

Email: [chhshi@foxmail.com](mailto:chhshi@foxmail.com)

Nengwu Zhu^1, 2*^

*Corresponding author

Email: [nwzhu@scut.edu.cn](mailto:nwzhu@scut.edu.cn)

Yanlan Cao^1^

Email: [237507619@qq.com](mailto:237507619@qq.com)

Pingxiao Wu^1, 2^

Email: [pppxwu@scut.edu.cn](mailto:pppxwu@scut.edu.cn)

^1^ School of Environment and Energy, South China University of Technology, Guangzhou 510006, China

^2^ The Key Laboratory of Pollution Control and Ecosystem Restoration in Industry Clusters of Ministry of Education, Guangzhou 510006, China


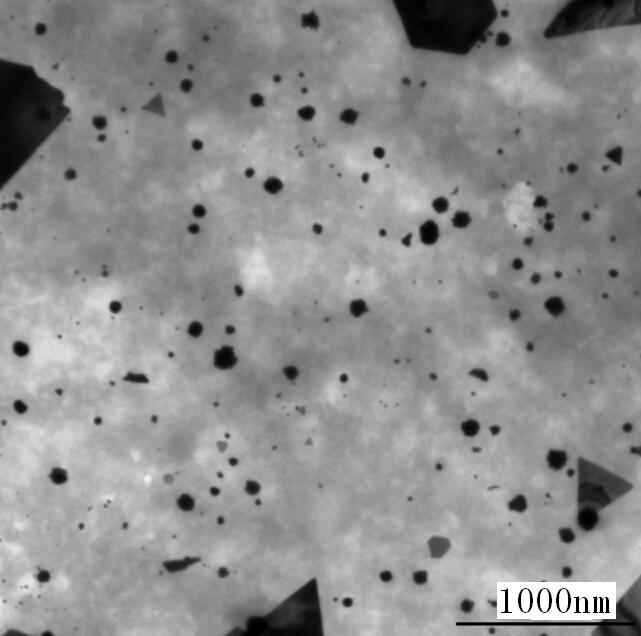


10ml IPE


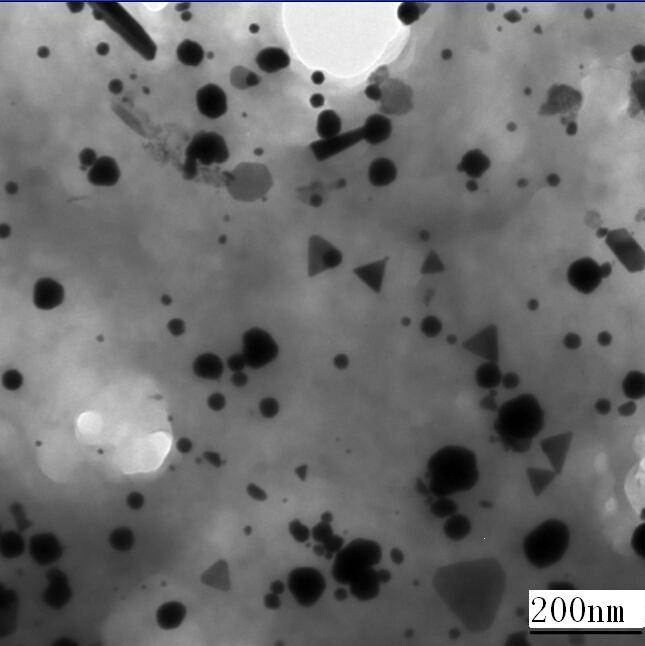


20ml IPE


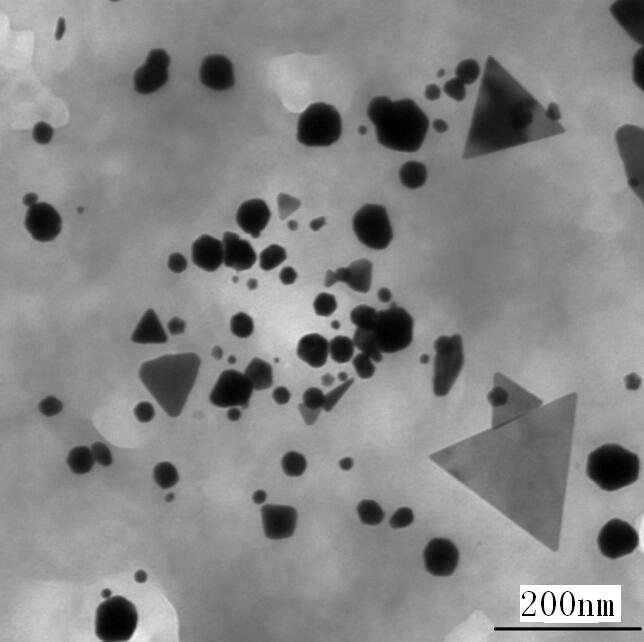


40ml IPE


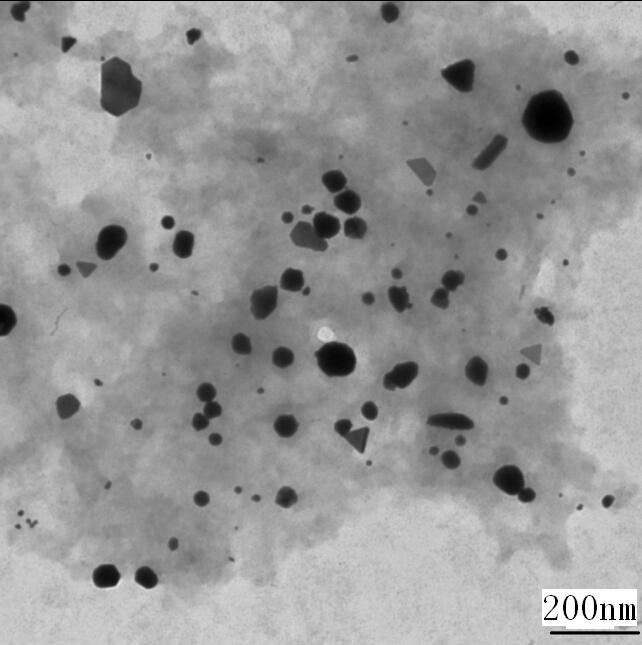


80ml IPE

Figure S1 TEM images of AuNPs synthesized with different IPE additions.


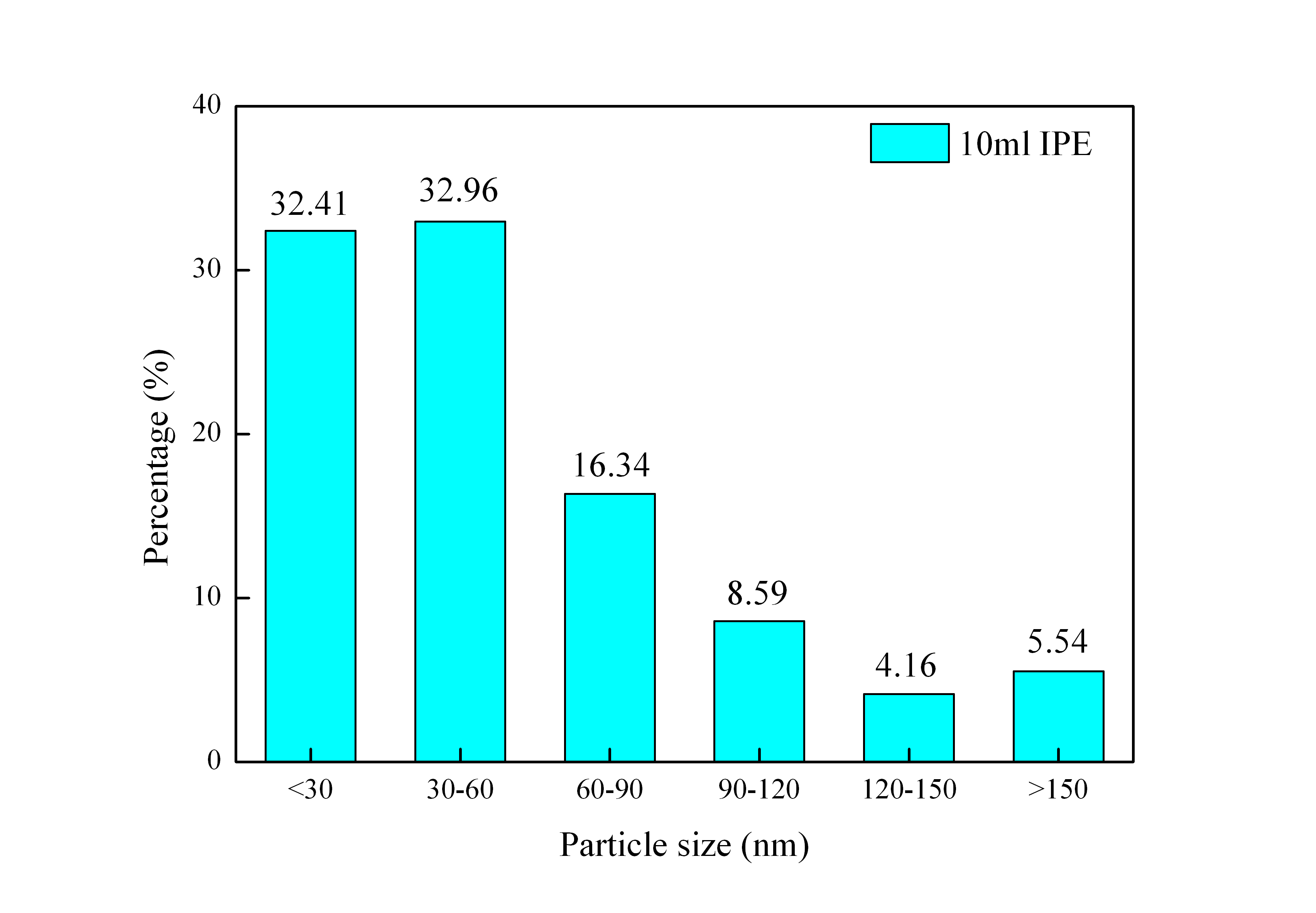

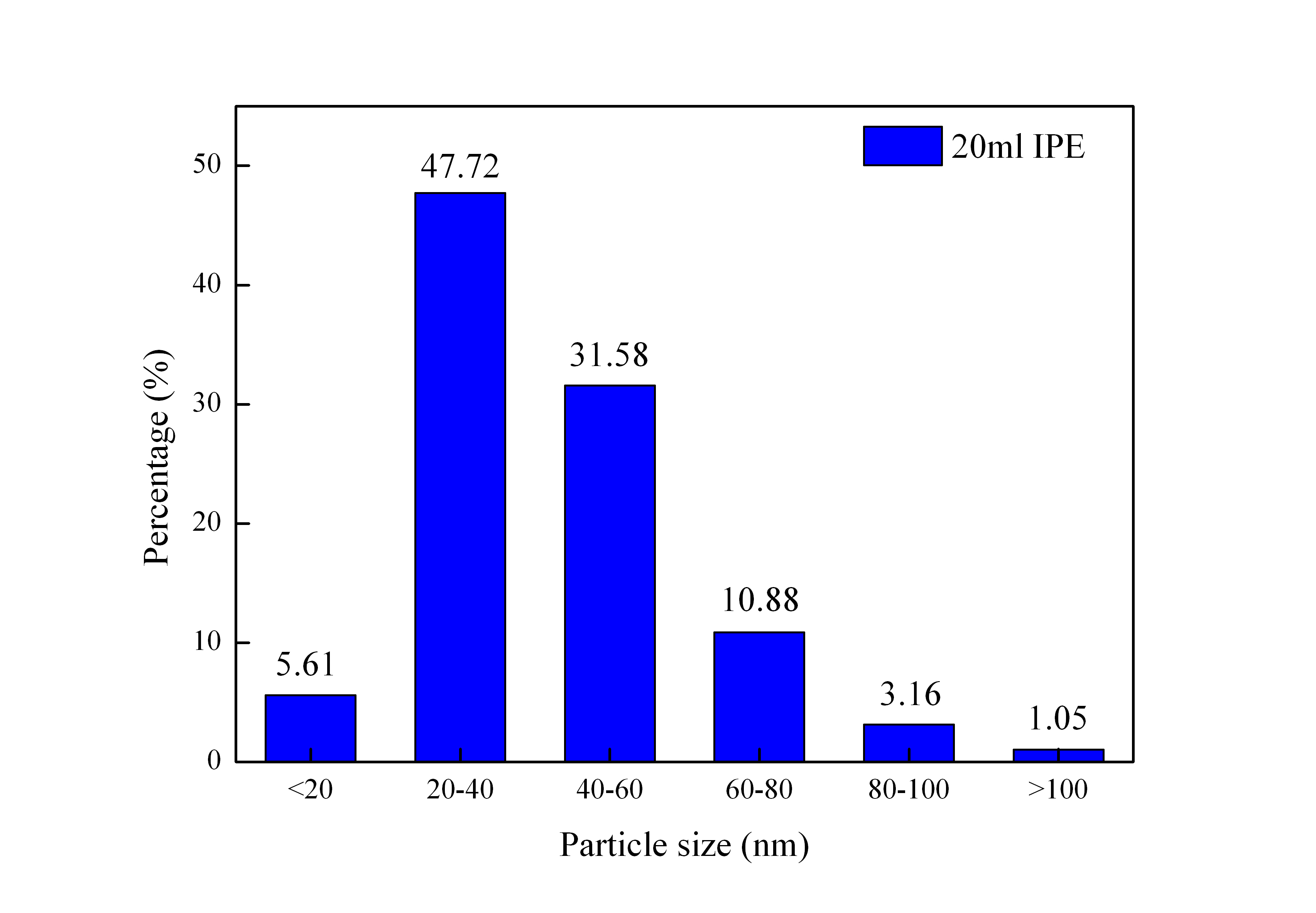

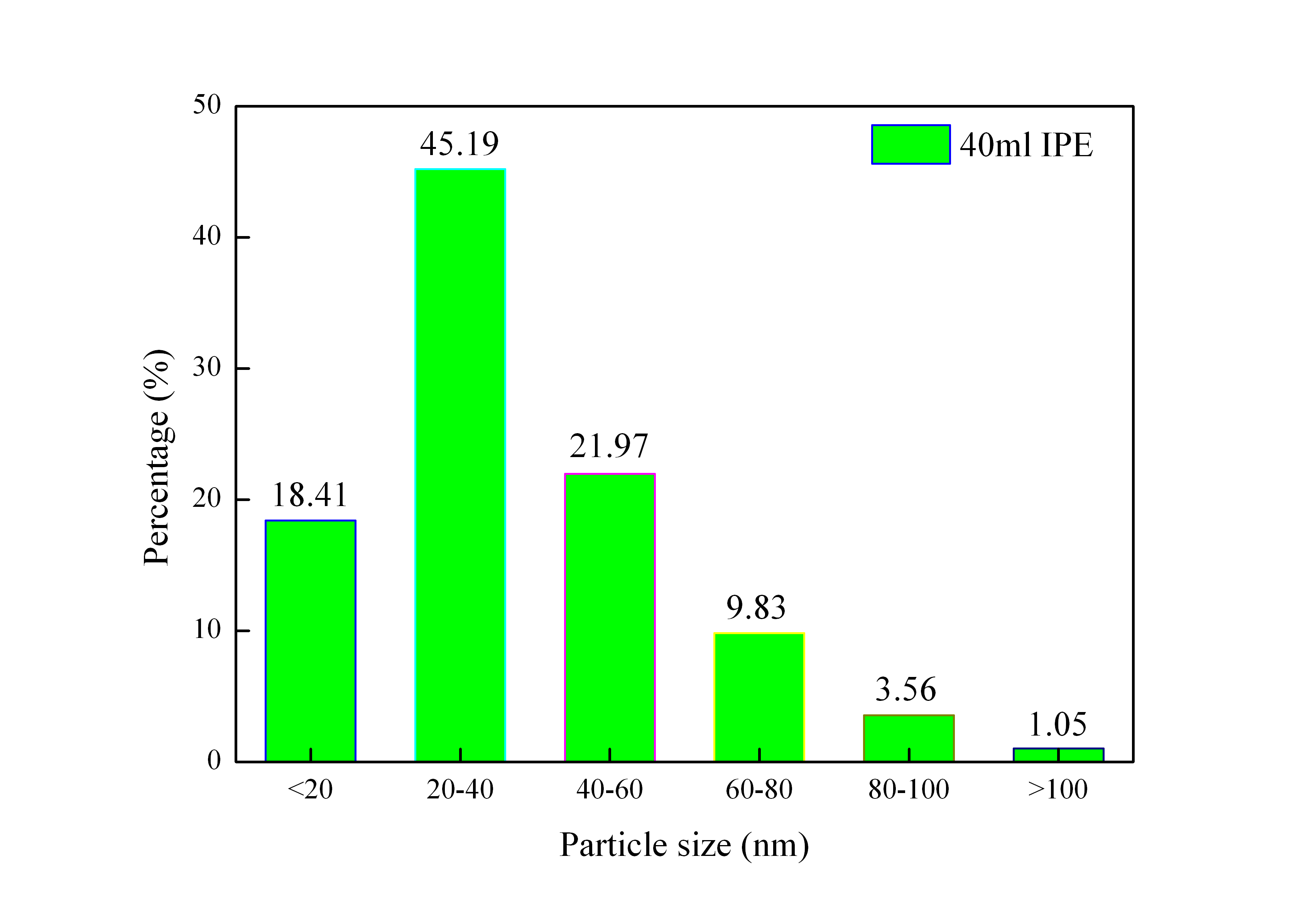

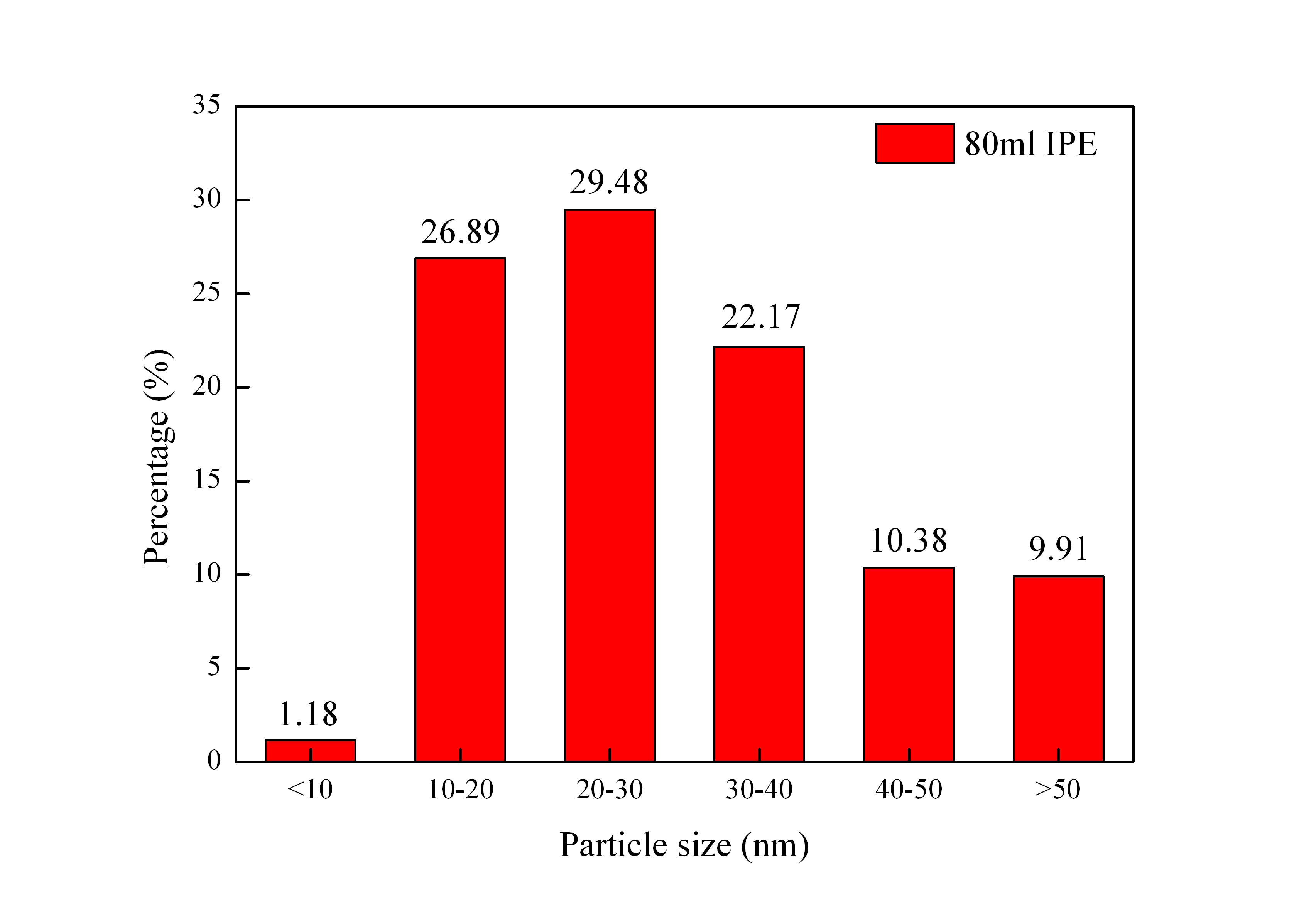


Figure S2 Particle size histograms of AuNPs synthesized with different IPE additions (measured more than 300 nanoparticles).


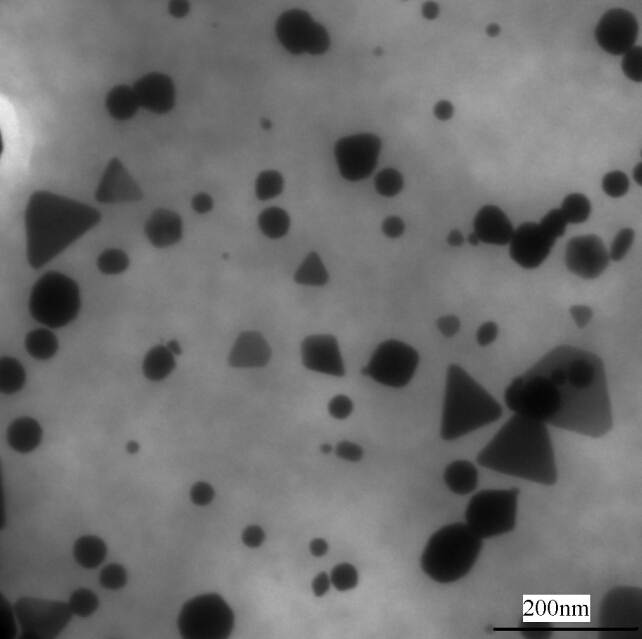


1.5mM


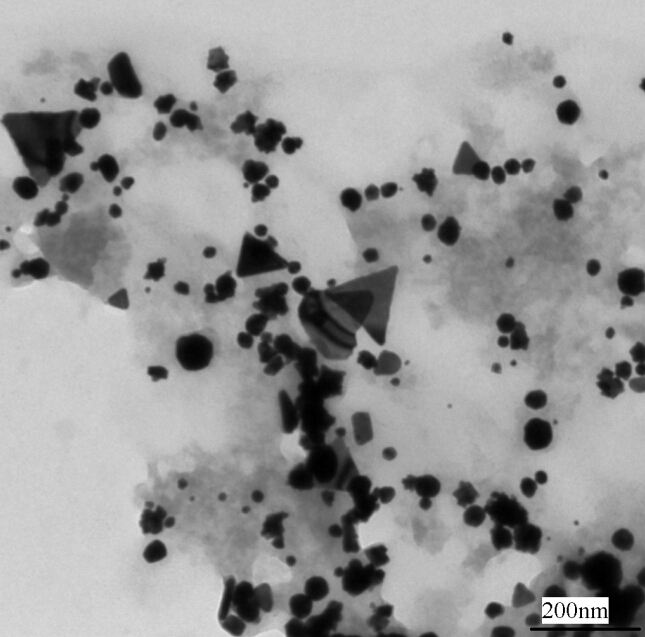


0.5mM


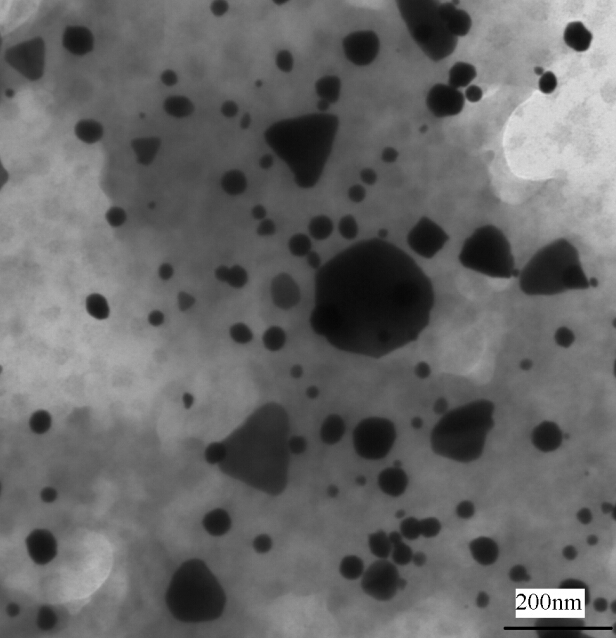


1.0mM


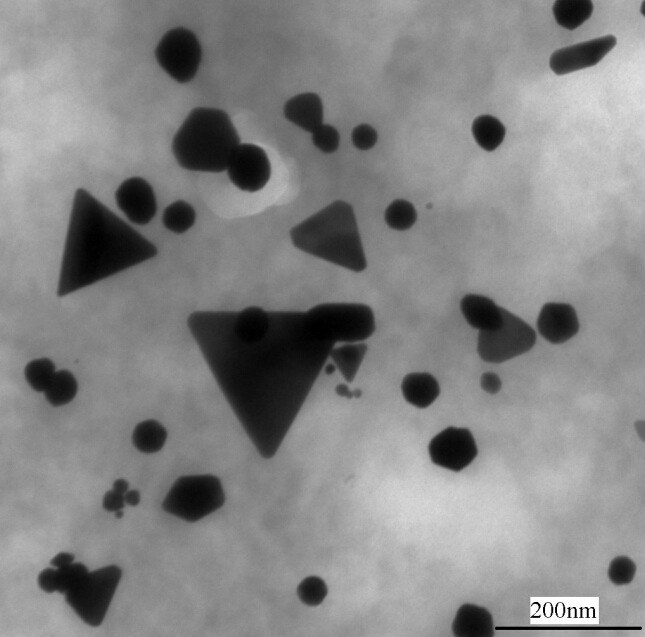


2.0mM

Figure S3 TEM images of AuNPs synthesized with different initial gold ion concentrations.


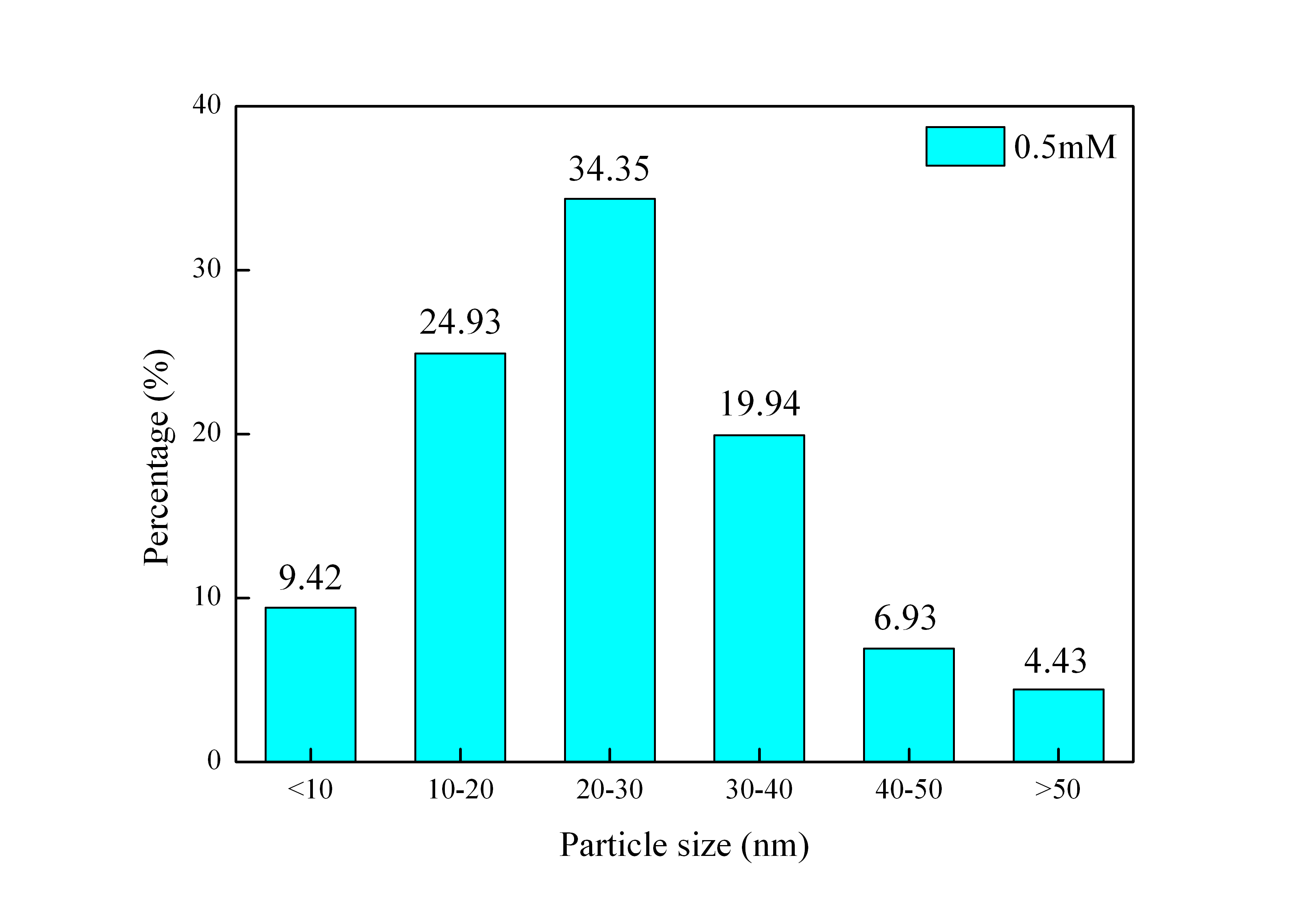

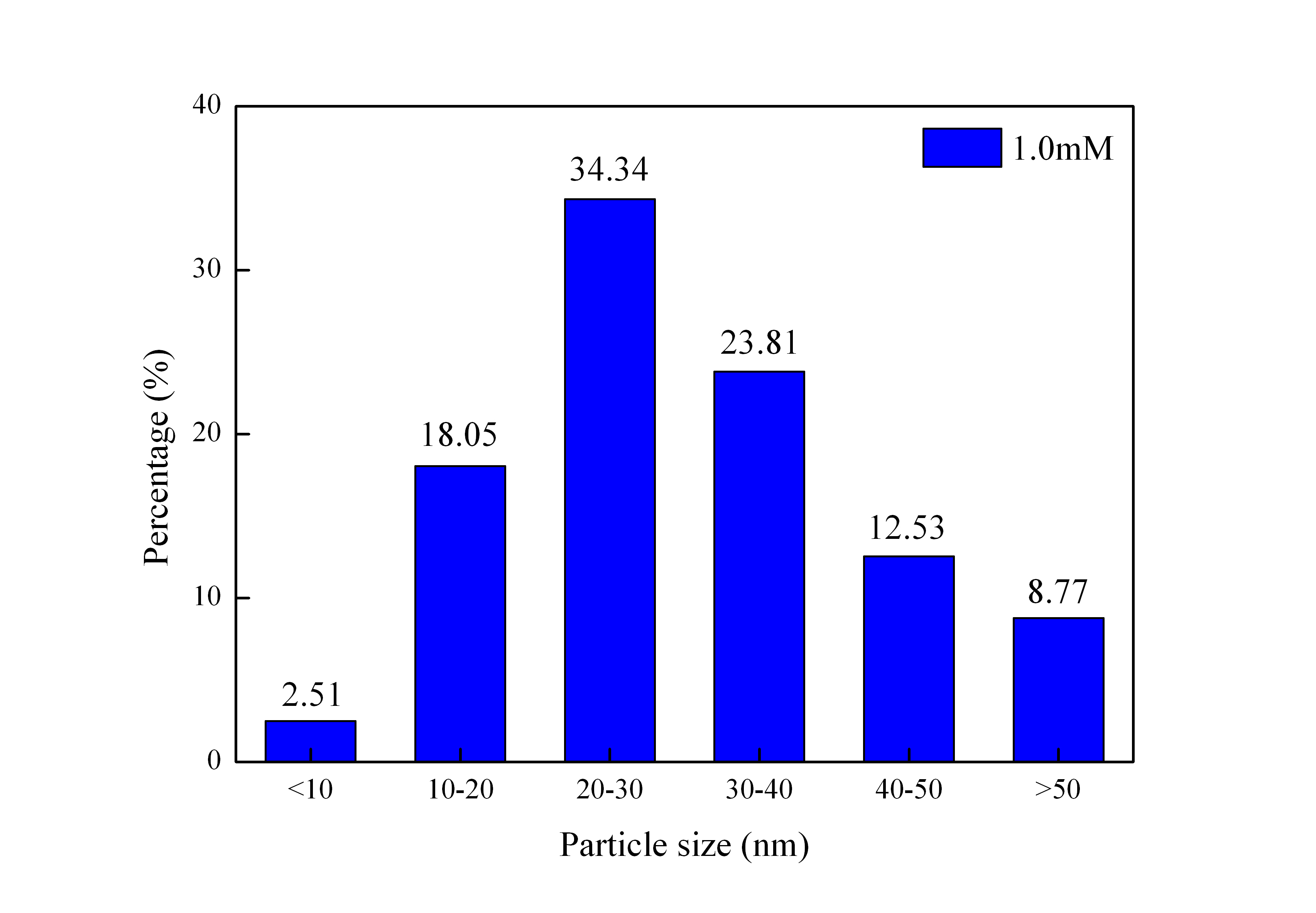

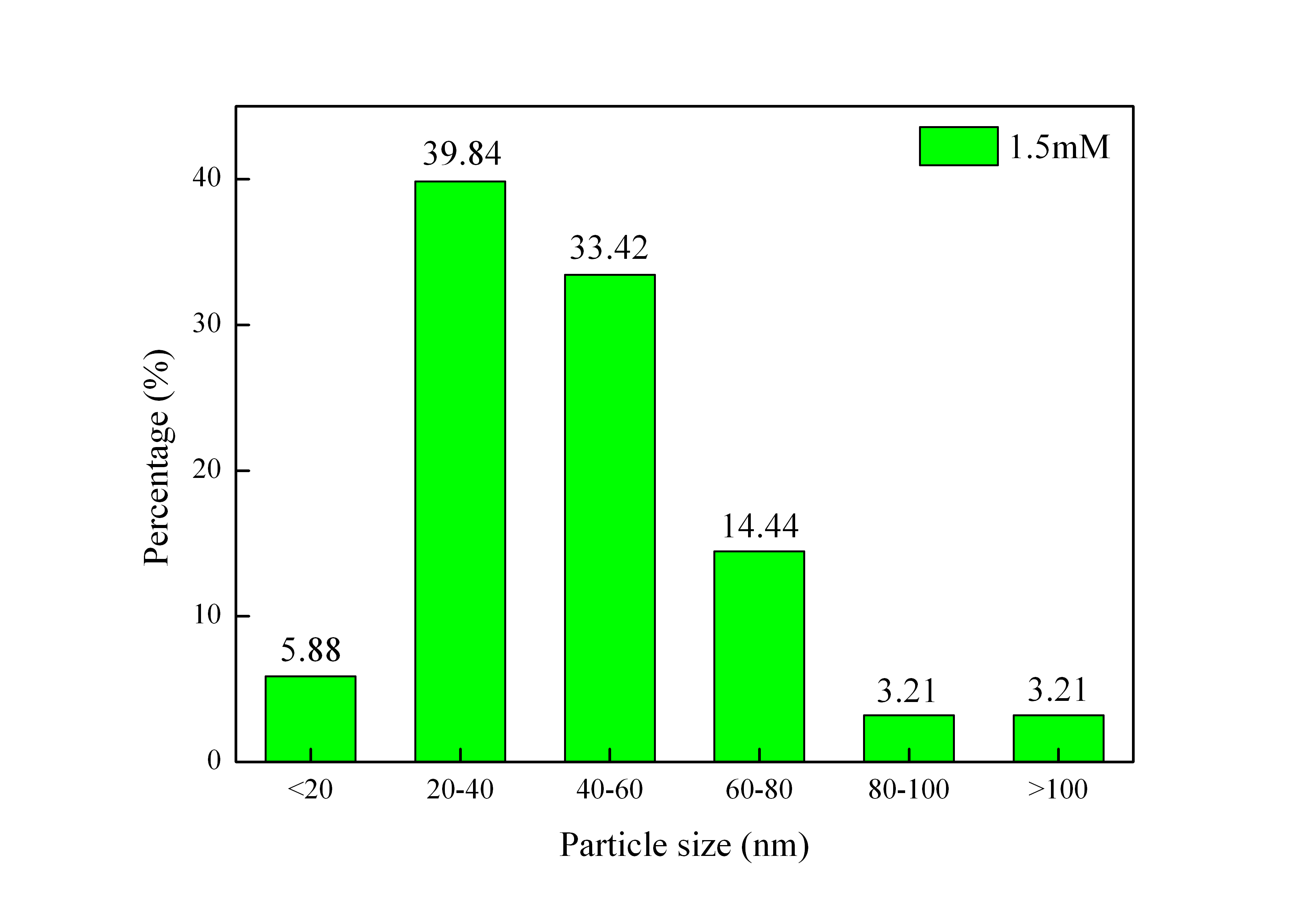

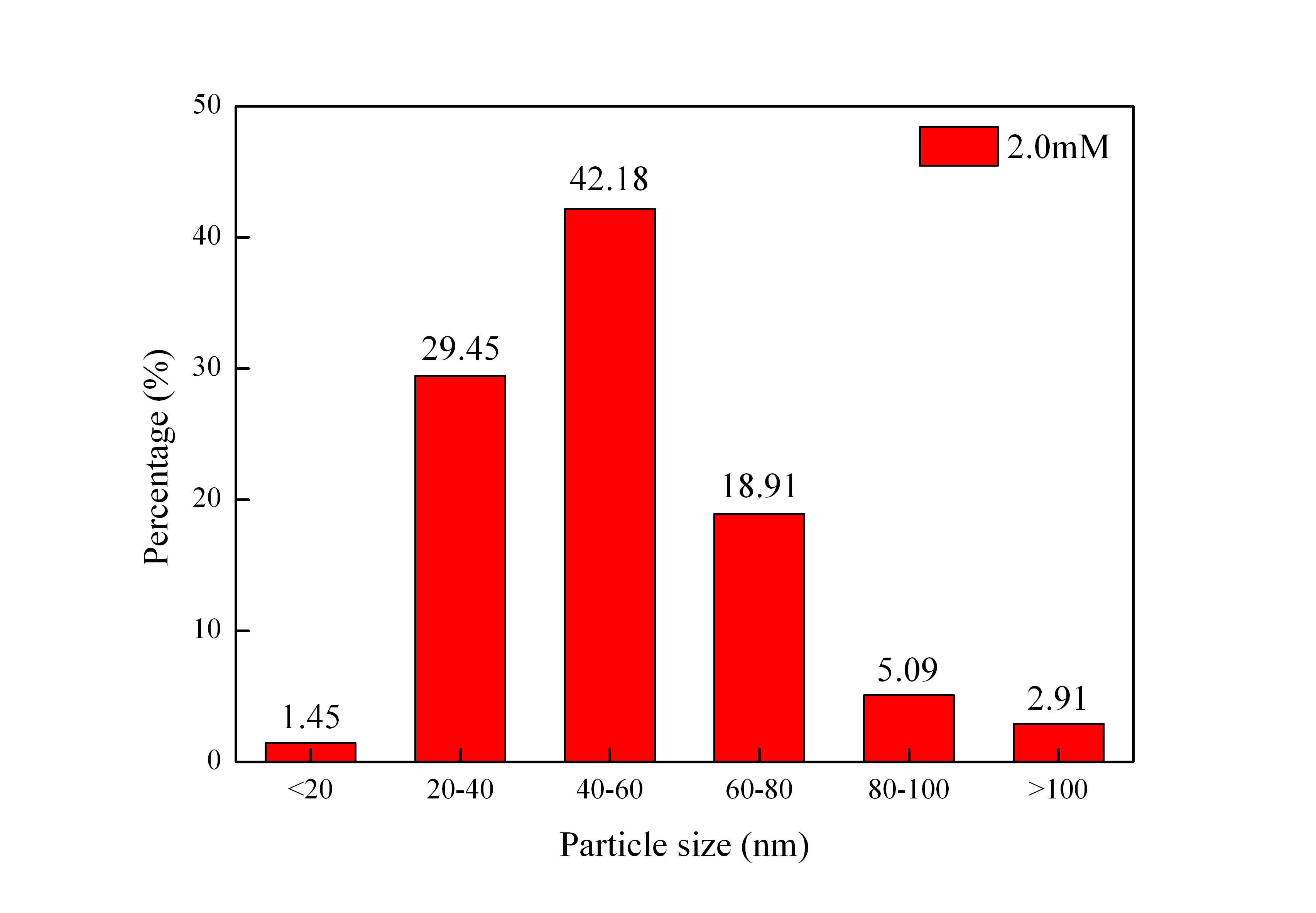


Figure S4 Particle size histograms of AuNPs synthesized with different initial gold ion concentrations (measured more than 300 nanoparticles).


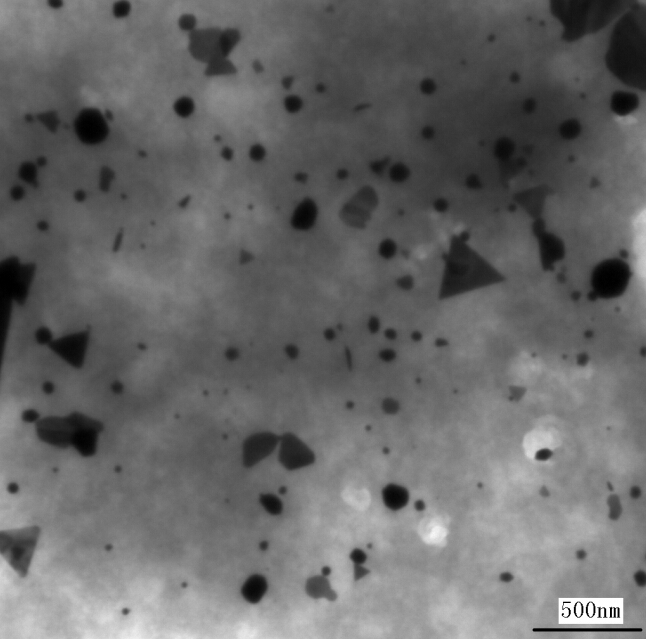


pH=2.0


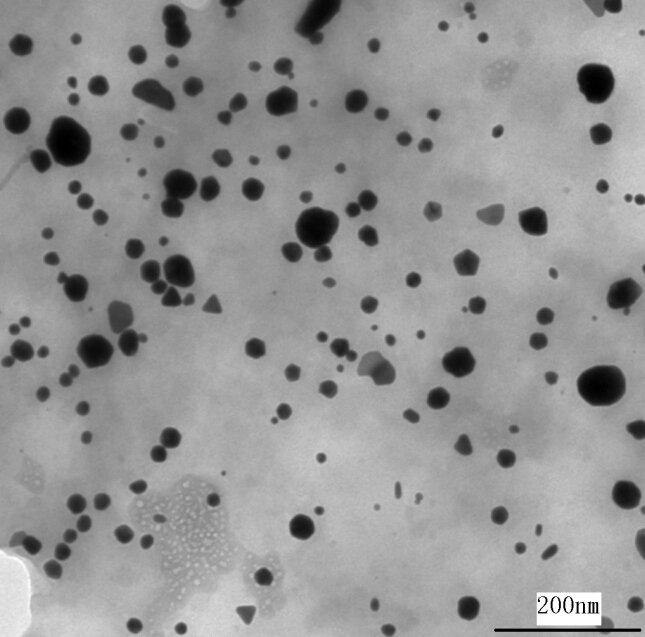


pH=4.0


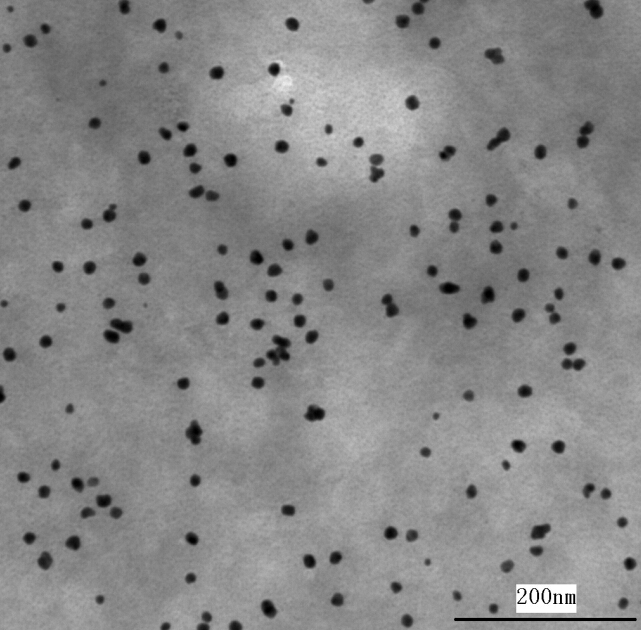


pH=6.0


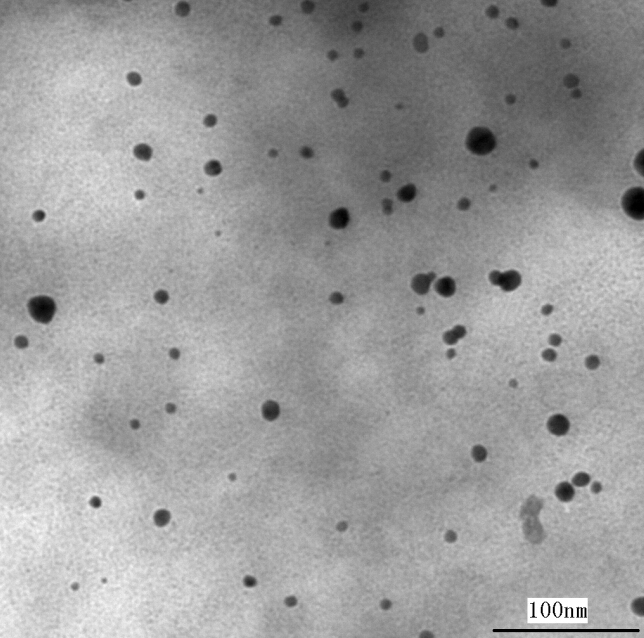


pH=8.0


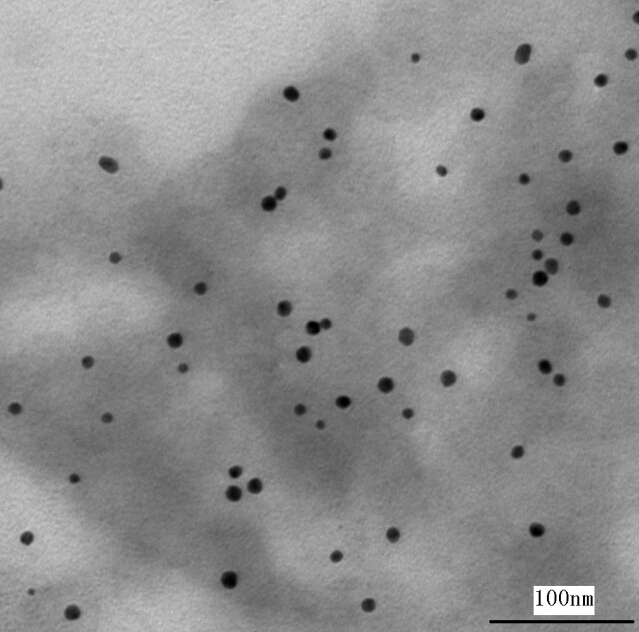


pH=10.0


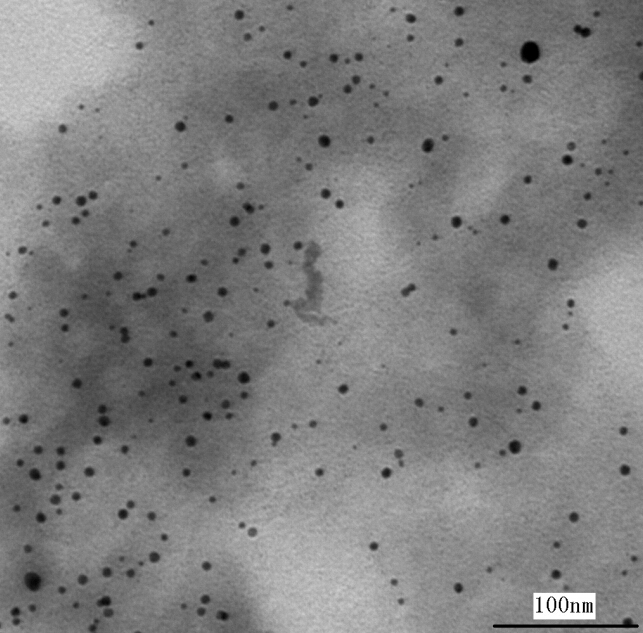


pH=12.0

Figure S5 TEM images of AuNPs synthesized with different initial solution pHs.


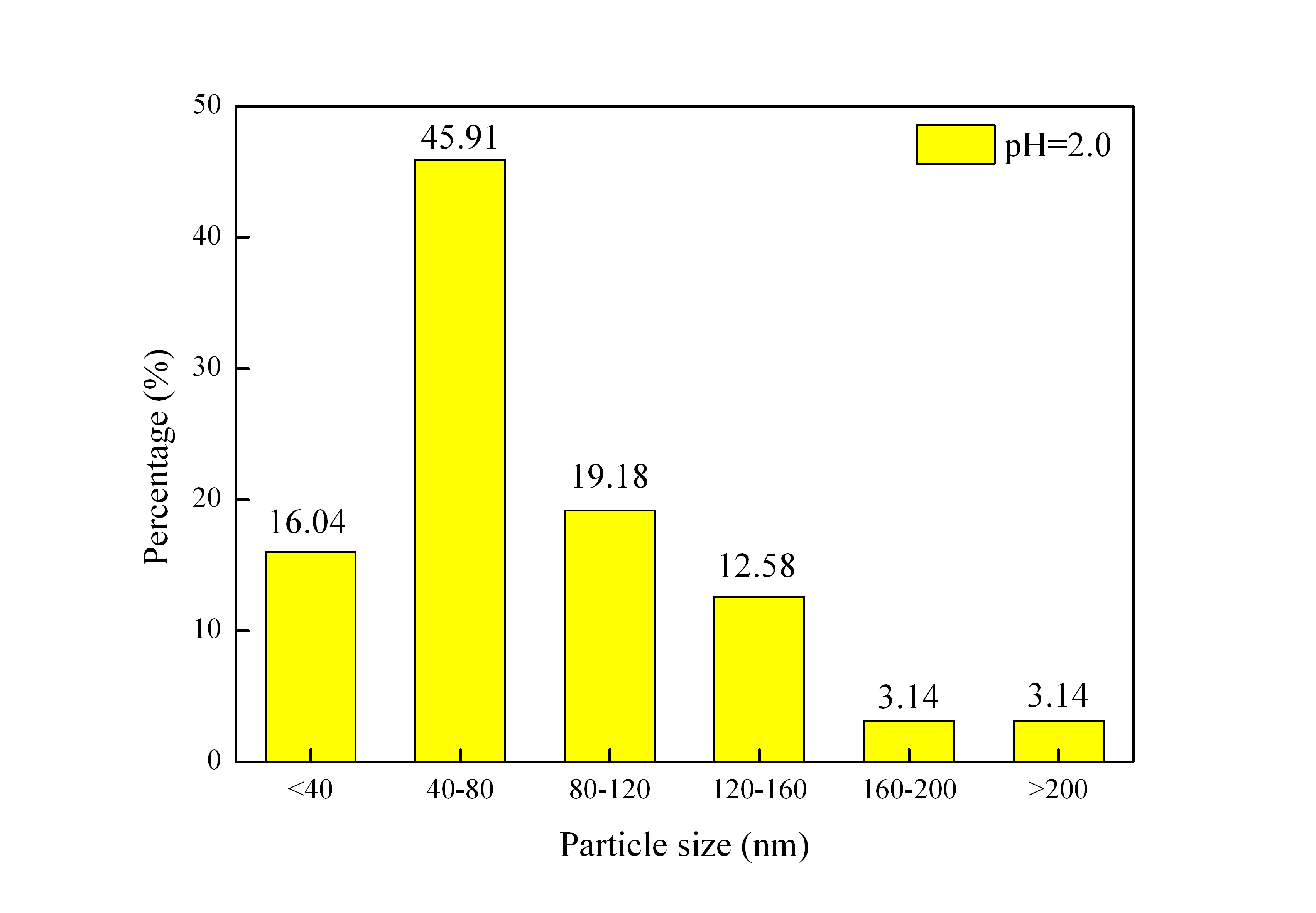

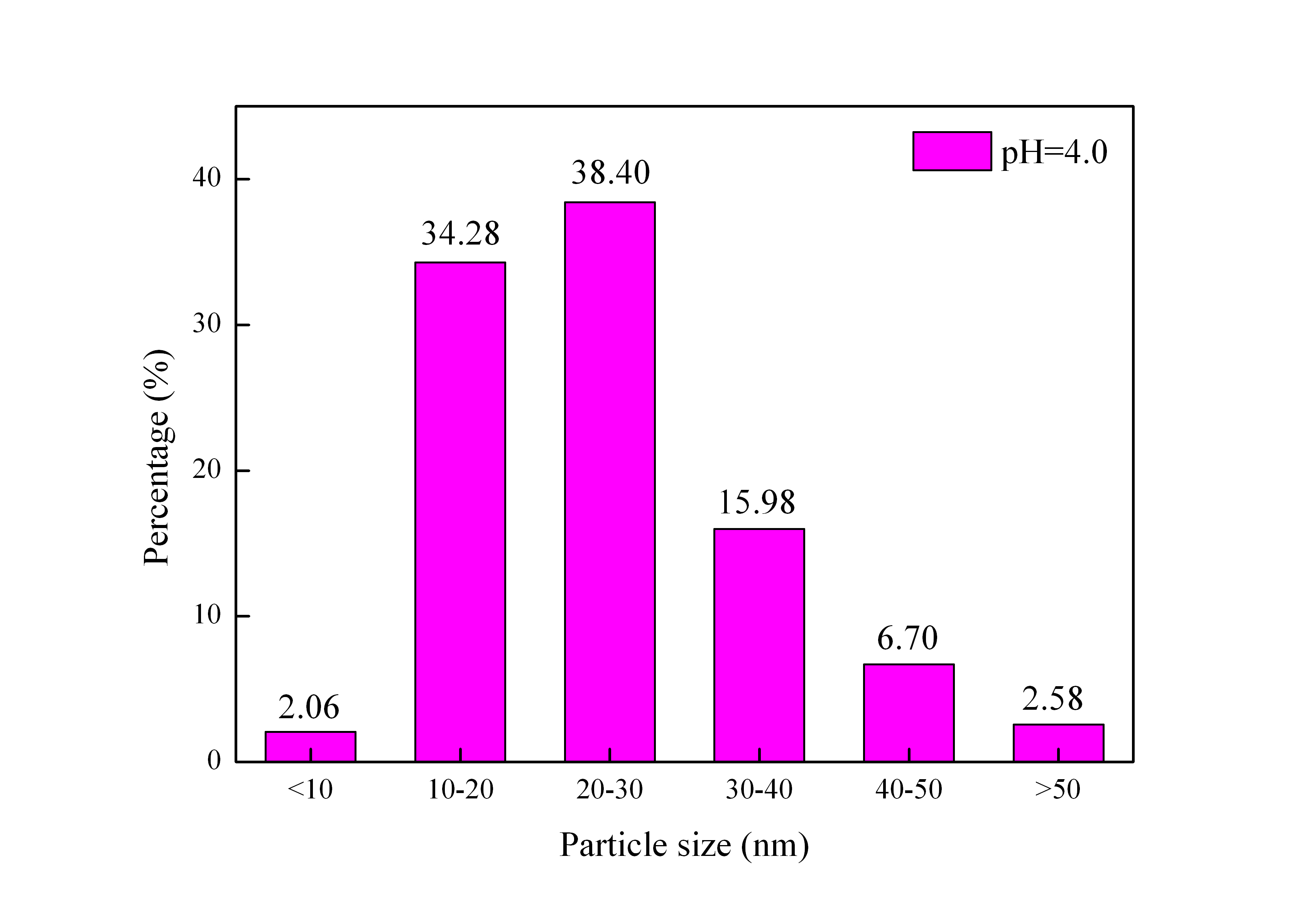

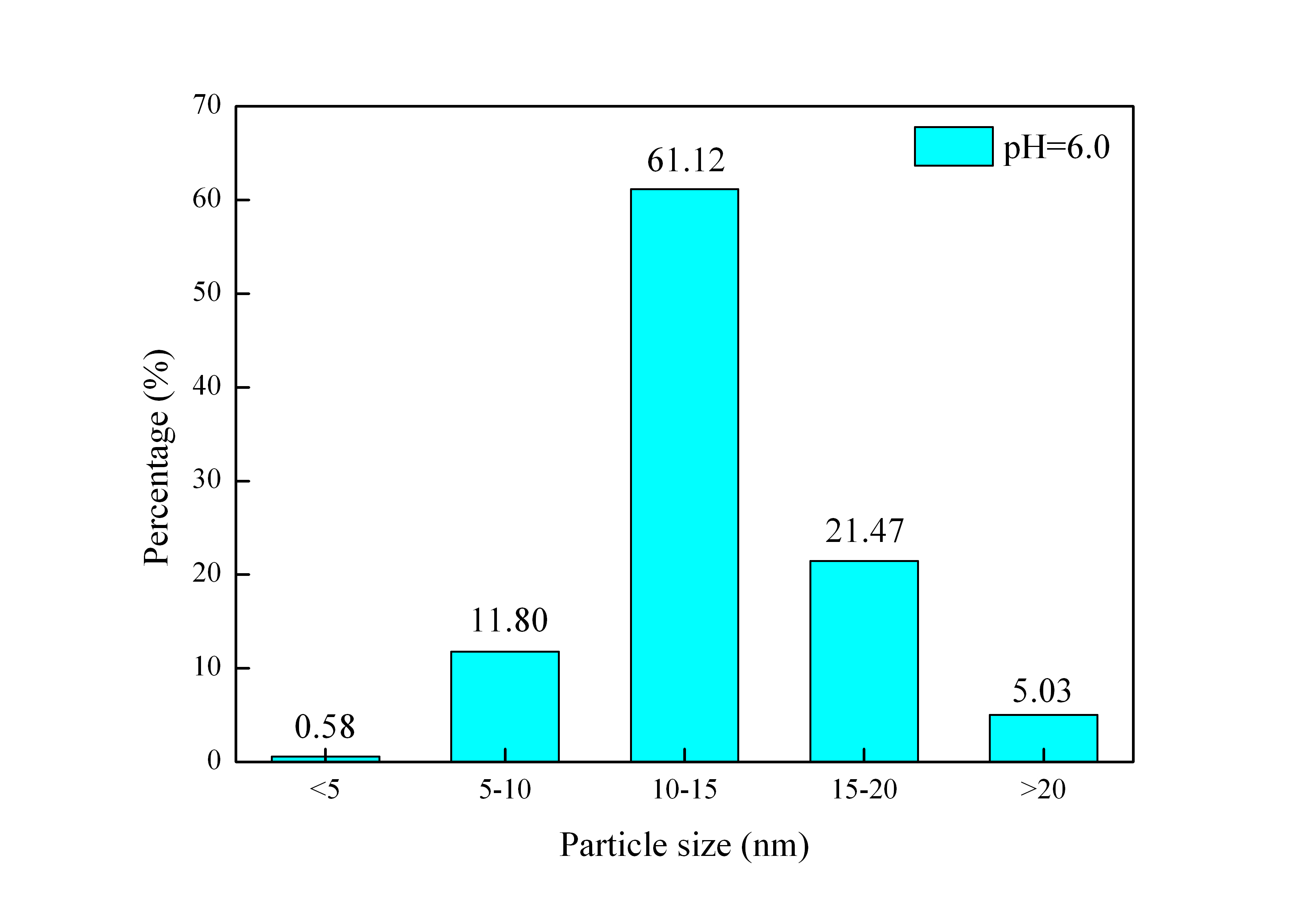

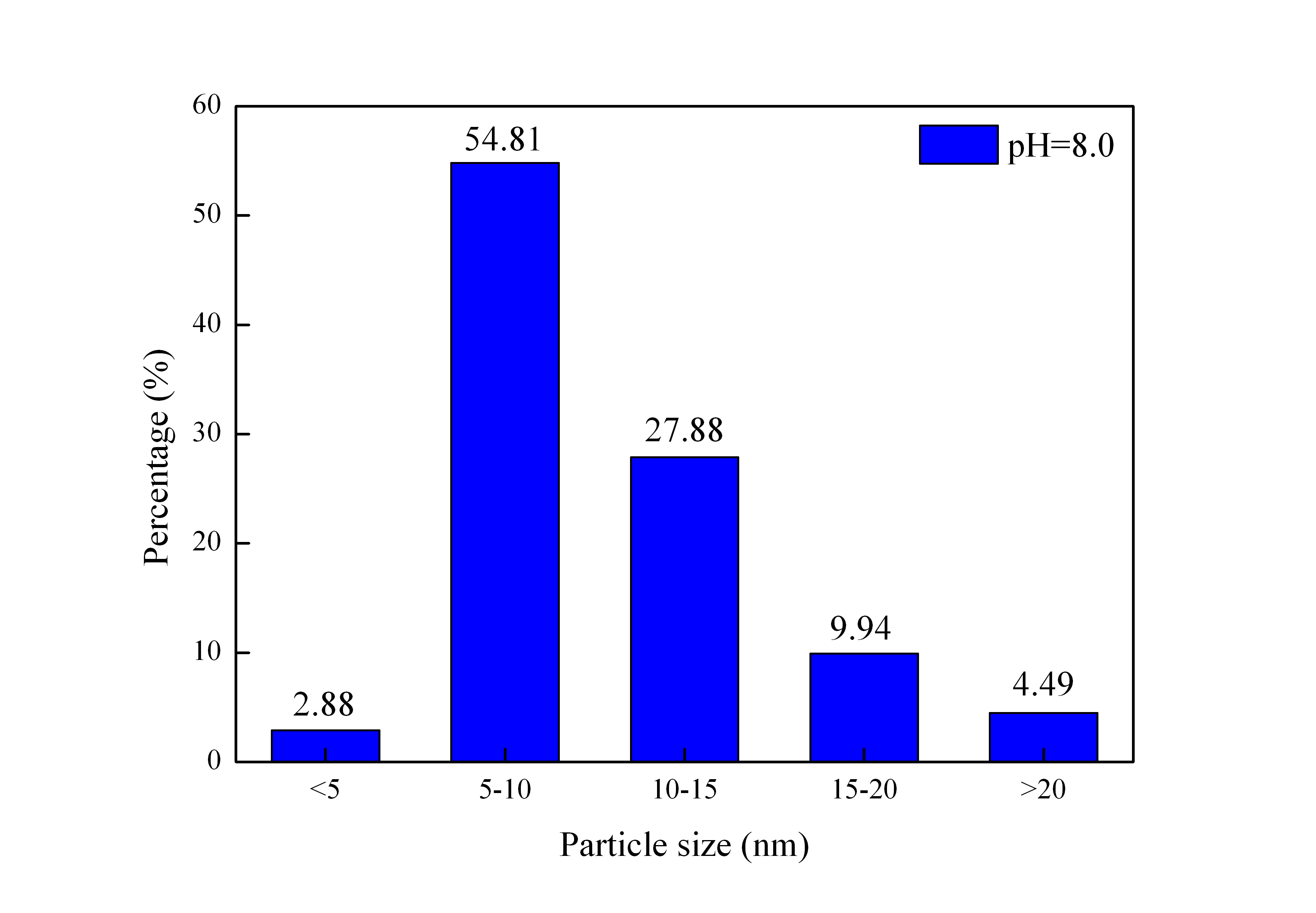

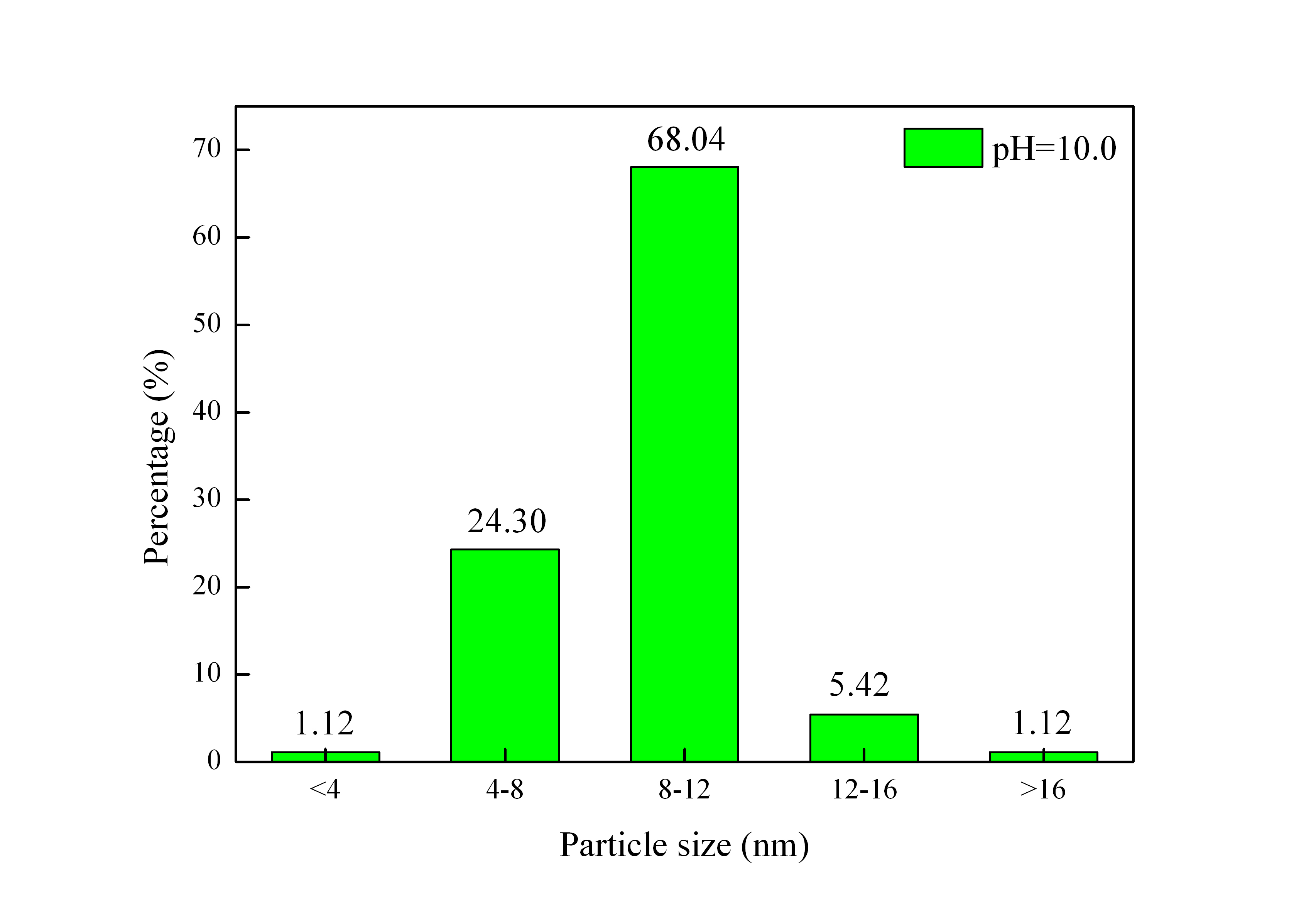

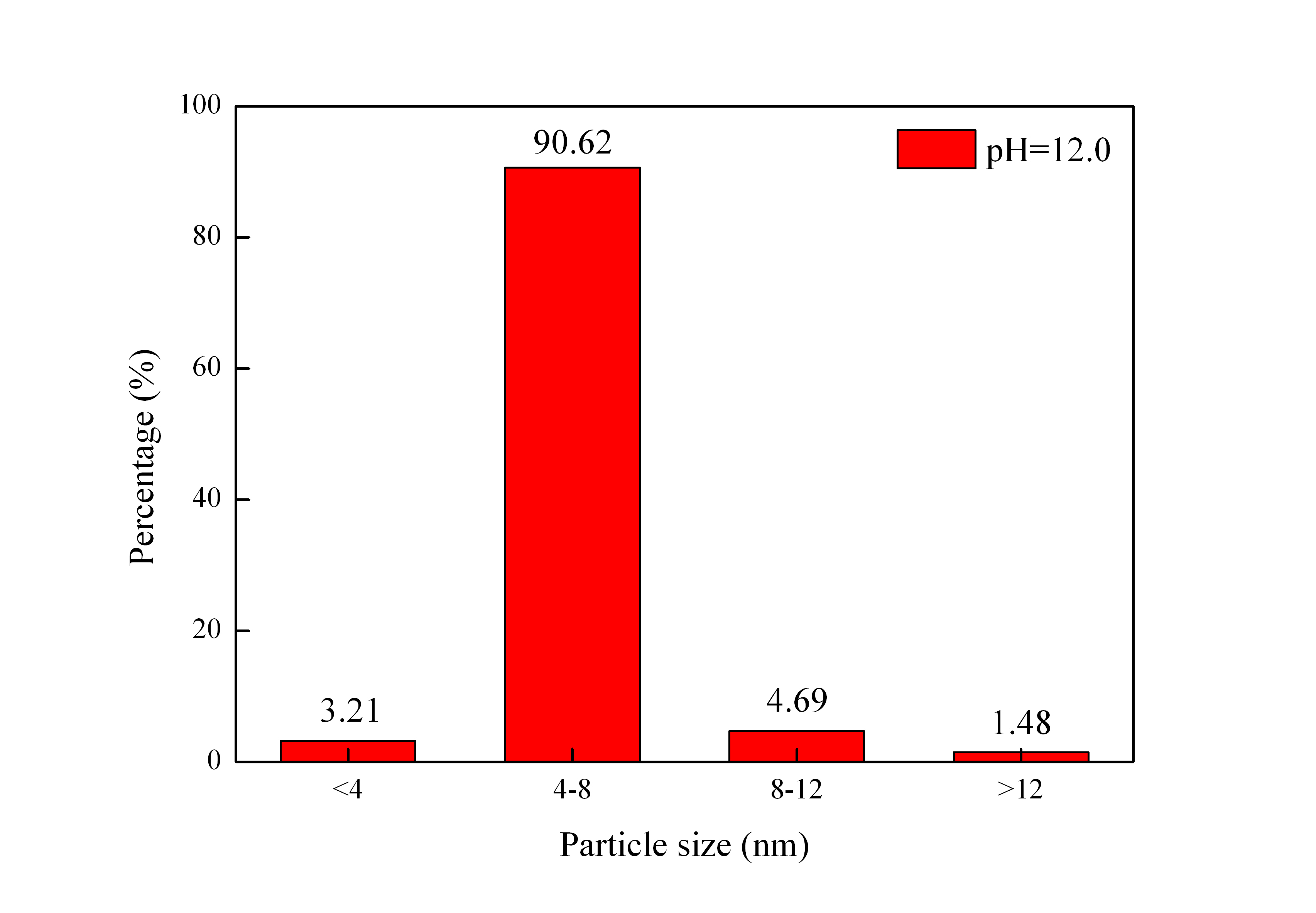


Figure S6 Particle size histograms of AuNPs synthesized with different initial solution pHs (measured more than 300 nanoparticles).
